# The Prevalence of Video Game Addiction and Its Relation to Anxiety, Depression, and Attention Deficit Hyperactivity Disorder (ADHD) in Children and Adolescents in Saudi Arabia: A Cross-Sectional Study

**DOI:** 10.7759/cureus.42957

**Published:** 2023-08-04

**Authors:** Nader Alrahili, Mohammad Alreefi, Issa M Alkhonain, Malak Aldakhilallah, Jamal Alothaim, Abdulwahed Alzahrani, Abdulrahman Alshargi, Nuran Baabbad

**Affiliations:** 1 Psychiatry, Imam Mohammad Ibn Saud Islamic University, Riyadh, SAU; 2 Pediatrics, King Abdullah Pediatric Hospital, Riyadh, SAU; 3 Family Medicine, Imam Mohammad Ibn Saud Islamic University, Riyadh, SAU; 4 Psychiatry, College of Medicine, Imam Mohammad Ibn Saud Islamic University, Riyadh, SAU

**Keywords:** adolescent, children, depression, anxiety, adhd, video game addiction

## Abstract

Background and objective

Video games have become a popular source of entertainment among children and adolescents, not only targeting the young generation but also increasingly popular among older demographics as well. This study aimed to assess the association between video game addiction and depression, anxiety, and attention deficit hyperactivity disorder (ADHD) among children and adolescents in Saudi Arabia.

Materials and methods

This was a cross-sectional study involving Saudi adolescents aged 12-16 years. A self-administered online survey was distributed on social media (Twitter, WhatsApp, and Snapchat). The survey addressed sociodemographic characteristics (i.e., age, gender, area of residence city, etc.). Arabic versions of the Patient Health Questionnaire-9 (PHQ-9), the seven-item Generalized Anxiety Disorder Scale (PHQ-GAD7), the ADHD Rating Scale-IV (ADHD-RS), and the seven-item Game Addiction Scale (GAS-7) questionnaires were distributed as well, including Arabic terms for depression, anxiety, and ADHD.

Results

A total of 393 surveys were completed (males: 58.3% vs. females: 41.7%). Of note, 63.1% of the respondents reported playing video games every day with an average of more than five hours of gaming time per day (25.2%). The prevalence of video game addiction was 62.1%. Independent risk factors of video game addiction included being young in age, playing video games every day, playing more than three hours per day, and playing multiplayer games. Furthermore, our findings suggest that a higher video game addiction score is correlated with higher scores in inattention, anxiety, and depression.

Conclusion

This study found a high prevalence of video game addiction among Saudi children and adolescents. Excessive video game playing negatively influences mental health and leads to issues including anxiety, depression, and ADHD. Also, younger males with an increased rate of regular playtime were more likely to exhibit video game addiction in comparison to the rest of the population. Further research is required to more comprehensively assess the prevalence rate of video game addiction and its effect on the mental health of the younger generation within the region.

## Introduction

Video games, whether online or offline, can be a source of addiction for adolescents. Studies by the American Psychiatric Association (APA) and the World Health Organization (WHO) have shown that they are related to mental health conditions in children and adolescents. The WHO labels it as "gaming disorder" and has defined it as impaired control over gaming, increasing priority given to gaming over other activities to the extent that gaming takes precedence over other interests and daily activities, and continuation or escalation of gaming time despite the occurrence of negative consequences. Current estimates suggest that video game addiction ranges between 1 and 15% in Europe and 3 and 8.5% in the US, while it is as high as approximately 14% in the UK and Korea and 17% in Iran; of note, 11.4% of the adolescents in Saudi Arabia are considered to be under a high level of stress [1,2,3,4].

It has been recognized that there is some correlation between gaming addiction and poor mental health conditions, mainly anxiety, depression, and inattention issues. Stress and anxiety affect the life of a child or adolescent in several ways; they affect their health, familial relationships, educational performance, social life, and ability to learn new things and make new friends. While there is some data on anxiety and stress among Saudi adolescents, scarce data is available on video game addiction. A study was conducted in Al-Qassim with a view to shed light on video game addiction and perceived stress among Saudi adolescents. Gaming addiction was measured with the Game Addiction Scale (GAS), while stress was measured with the Perceived Stress Scale (PSS). The researchers found that nearly 75% of the participants experienced stress, including 11.4% who had high levels of stress. Around 5% of the participants were addicted to gaming. The study also reported that gaming addiction is associated with higher levels of perceived stress in Saudi adolescents, which raises concerns about the mental health of Saudi youth [1].

Another study was conducted to observe the impact of video game addiction and psychological distress among expatriate adolescents. It indicated that non-Saudi adolescents who live in Saudi Arabia may have an increased risk for video game addiction, which is strongly associated with greater distress. The study recommended that public health authorities and researchers prioritize examining the effects of video game use and addiction in the community. Future research should aim to gain a more comprehensive understanding of video game addiction among adolescents in Saudi Arabia for the development of programs to manage this problem and its consequences [2]. 

The prevalence of depression has been on the rise in the last decade and has become a leading cause of disability in recent years globally. In the US, childhood depression is associated with a significant economic burden on the healthcare system. The prevalence of depression among children aged 3-17 years was 4.9% across the US in 2012. In Saudi Arabia, there is limited data to help estimate the extent of depression among children. A study conducted in 2019 in Riyadh focused on adolescents aged between 12-19 years and showed that out of 960 students, 32.4% had moderate to severe depression [3]. Another study conducted in Dammam showed that the prevalence of depression among high school male students with internet gaming disorder was 21.7%, including those with moderate to severe depression [4].

Attention deficit hyperactivity disorder (ADHD) is a neurodevelopmental disorder characterized by a lack of attention, aggressiveness, and/or hyperactivity, and is primarily diagnosed in childhood [5]. The prevalence of ADHD among children and adolescents is reported to be 5-9% (average: 7%) and 3-5% in adults, with a global prevalence of 5% [6]. Due to their neuropsychological profile, children with ADHD are particularly prone to the compulsive use of computer games. Furthermore, in the setting of psychological factors associated with violent thoughts and behaviors, playing computer games that feature excessive violence may be a substantial risk factor for aggressive conduct [7]. However, more studies on this topic are required in Saudi Arabia because gaming among children and adolescents is becoming more common in the country and there is currently limited data pertaining to gaming addiction and its correlation with various mental health conditions. This study aims to measure the prevalence of gaming addiction and its correlation with depression, anxiety, and ADHD among children in Saudi Arabia.

## Materials and methods

This was a cross-sectional study conducted to evaluate the association of gaming addiction with depression, anxiety, and inattention among children and adolescents in Saudi Arabia. We employed a validated Arabic version of the Patient Health Questionnaire-9 (PHQ-9) [8,9] to measure depressive symptoms, a validated Arabic version of the seven-item Generalized Anxiety Disorder scale (PHQ-GAD7) [9,10] to assess anxiety symptoms, a validated Arabic version of the ADHD Rating Scale-IV (ADHD-RS) [11,12] to determine inattention, and a validated Arabic version of the seven-item Game Addiction Scale (GAS-7) [13,14] to determine the level of game addiction. In addition, eight questions were added pertaining to the types of games, hours of playing, and family supervision. The sample initially consisted of 414 participants, 21 of whom were excluded because they resided outside Saudi Arabia. The 393 participants who were ultimately included consisted of children and adolescents aged 12-16 years who lived in Saudi Arabia. A convenience sampling technique was used. Data were collected by distributing a link that redirected the participants to an integrated survey platform using Google Forms, which was shared through various social media platforms. The questionnaire was filled out by the children and adolescents with the guided assistance of a guardian. The inclusion criterion was children or adolescents aged from 12 to 16 years old. Exclusion criteria were non-Arabic-speaking children and adolescents, those with non-Arabic-speaking guardians, those residing outside Saudi Arabia as well as children or adolescents with intellectual disability.

Statistical analysis

Data were summarized using numbers, percentages, means, and standard deviation (SD). The relationship between the level of video game addiction and sociodemographic characteristics and the frequency of playing games was analyzed using the Chi-square test. A multivariate regression model was used to determine the independent significant factors associated with game addiction with the corresponding odds ratio (OR) as well as a 95% confidence interval (CI). The Pearson correlation coefficient was also used to determine the correlation between the score of video game addiction and ADHD, anxiety, and depression. A p-value of 0.05 was considered statistically significant, while a p-value of 0.01 was considered highly statistically significant. All statistical analyses were carried out using IBM SPSS Statistics for Windows, Version 26.0 (IBM Corp., Armonk, NY).

## Results

A total of 393 children and adolescents completed the survey. As seen in Table 1, the most common age group was those aged 16 years (36.4%), with nearly 60% being male. Nearly half of them (49.6%) were living in Riyadh. Parents mostly filled out the questionnaire (37.9%) followed by the child or adolescent (37.2%). Most parents were living in the same house (88.5%) as their children. Regarding the educational level of the father, 37.7% were bachelor’s degree holders, and so were 47.1% of the mothers.

**Table 1:** Sociodemographic characteristics of the study participants (n=393)

Variables	N (%)
Age group	
12 years	114 (29.0%)
13 years	42 (10.7%)
14 years	48 (12.2%)
15 years	46 (11.7%)
16 years	143 (36.4%)
Gender	
Male	229 (58.3%)
Female	164 (41.7%)
City of residence	
Riyadh	195 (49.6%)
Jeddah	27 (6.9%)
Makkah	16 (4.1%)
Al Madinah	34 (8.7%)
Dammam	11 (2.8%)
Al Khobar	08 (2.0%)
Al Kharj	16 (4.1%)
Jazan	10 (2.5%)
Al Qassim	19 (4.8%)
Taif	09 (2.3%)
Tabuk	09 (2.3%)
Others	39 (9.9%)
Who will fill out the questionnaire?	
Child or adolescent	146 (37.2%)
One of the parents	149 (37.9%)
One of the siblings	92 (23.4%)
Others	06 (1.5%)
Marital status of the parents	
Live in the same house	348 (88.5%)
Divorced	25 (6.4%)
One of them is dead	20 (5.1%)
Father's educational level	
Elementary school	31 (7.9%)
Intermediate school	29 (7.4%)
High school	98 (24.9%)
Bachelor's degree	148 (37.7%)
Postgraduate degree	87 (22.1%)
Mother's educational level	
Elementary school	31 (7.9%)
Intermediate school	34 (08.7%)
High school	95 (24.2%)
Bachelor's degree	185 (47.1%)
Postgraduate degree	48 (12.2%)

As shown in Table 2, 63.1% of the respondents reported playing video games every day, with a quarter of them (25.2%) accustomed to playing for more than five hours daily. The proportion of the respondents playing multiplayer games regularly was 89.6%. The most common type of games usually played by the subjects were shooter games (39.2%) and action or survival games (22.1%). Approximately 59.3% were used to playing alone in the room while 48.9% of the parents stated that they supervised their children during game time. Additionally, 26.2% were seen to mimic the games in real life, e.g., doing impulsive movements or using inappropriate language.

**Table 2:** Characteristics of children and adolescents with regard to playing video games (n=393)

Variables	N (%)
Number of days spent in a week playing video games	
One day	22 (5.6%)
Two days	29 (7.4%)
Three days	24 (6.1%)
Four days	27 (6.9%)
Five days	29 (7.4%)
Six days	14 (3.6%)
Every day	248 (63.1%)
Number of hours spent in a day playing video games	
Less than one hour	38 (9.7%)
One to two hours	64 (16.3%)
Two to three hours	68 (17.3%)
Three to four hours	71 (18.1%)
Four to five hours	53 (13.5%)
More than five hours	99 (25.2%)
Playing multiplayer games regularly (online games)	
Yes	352 (89.6%)
No	41 (10.4%)
Type of games usually played	
Shooter games	154 (39.2%)
Sport games	62 (15.8%)
Adventure games	87 (22.1%)
Action and survival games	32 (8.1%)
Fighting games	30 (7.6%)
Strategy games	13 (3.3%)
Simulation games	15 (3.8%)
Where do you play usually?	
Alone in the room	233 (59.3%)
In the presence of the family	160 (40.7%)
Do the parents supervise the games you play?	
Yes	192 (48.9%)
No	201 (51.1%)
Does the child or adolescent mimic the games in real life like doing impulsive movements or saying bad words?	
Yes	103 (26.2%)
No	290 (73.8%)

**Figure 1:**
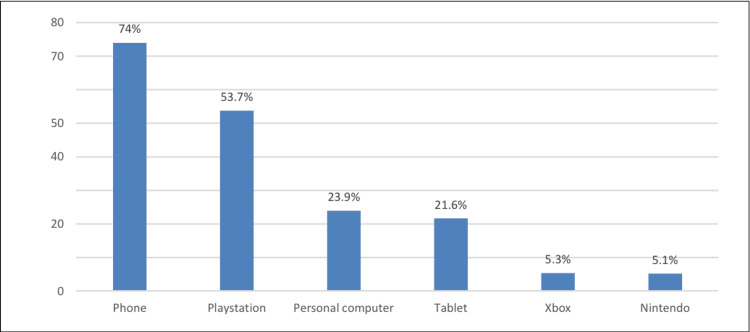
Common gaming platforms used

As depicted in Figure 1, the most common gaming platform used by the subjects was the phone (74%), followed by the PlayStation (53.7%) and personal computer (23.9%).

As summarized in Table 3, 62.1% of our responders reported addiction to video games; moreover, 20.9% reported severe anxiety and 14.2% reported experiencing severe depression.

**Table 3:** Descriptive statistics related to game addiction, ADHD, anxiety, and depression (n=393) ADHD: attention deficit hyperactivity disorder; SD: standard deviation

Variables	Values
Game addiction score, mean ± SD	4.01 ± 2.23
Level of game addiction, n (%)	
Addicted	244 (62.1%)
Not addicted	149 (37.9%)
ADHD total score, mean ± SD	16.2 ± 10.4
Inattention domain, mean ± SD	8.59 ± 5.50
Impulsivity or hyperactivity domain, mean ± SD	7.57 ± 5.40
Anxiety score, mean ± SD	8.56 ± 6.23
Severity of anxiety, n (%)	
No	124 (31.6%)
Mild	114 (29.0%)
Moderate	73 (18.6%)
Severe	82 (20.9%)
Depression score, mean ± SD	9.76 ± 7.55
Severity of depression, n (%)	
Minimal	119 (30.3%)
Mild	104 (26.5%)
Moderate	67 (17.0%)
Moderately severe	47 (12.0%)
Severe	56 (14.2%)

As presented in Table 4, the Pearson correlation indicated that there was a positive significant correlation observed between game addiction scores and ADHD (r=0.527), anxiety (r=0.366), and depression (r=0.321).

**Table 4:** Correlation (Pearson-r) between game addiction and ADHD, anxiety, and depression (n=393) *Correlation is significant at the 0.01 level (two-tailed) ADHD: attention deficit hyperactivity disorder

Variables	Game addiction score	
	R-value	P-value
ADHD	0.527	<0.001*
Anxiety	0.366	<0.001*
Depression	0.321	<0.001*

When assessing the relationship between game addiction and the sociodemographic characteristics and the frequency of playing video games (Table 5), the prevalence of game addiction was significantly more commonly associated with the younger age group (p=0.018), male gender (p<0.001), PlayStation gaming platform (p<0.001), playing every day (p<0.001), playing for more than three hours per day (p<0.001), playing shooter games (p=0.024), playing video games alone in the room (p=0.009), and mimicking the games in real life (p=0.002).

**Table 5:** Relationship between game addiction and sociodemographic characteristics and the frequency of playing games using an electronic device (n=393) ^†^Variable with multiple responses. ^§^P-value has been calculated using the Chi-square test. *Significant at p<0.05 level

Factor	Level of game addiction		P-value^§^
	Addicted, n (%) (n=244)	Not addicted, n (%) (n=149)	
Age group			
12–13 years	108 (44.3%)	48 (32.2%)	0.018*
14–16 years	136 (55.7%)	101 (67.8%)	
Gender			
Male	162 (66.4%)	67 (45.0%)	<0.001*
Female	82 (33.6%)	82 (55.0%)	
Area of residence			
Inside Riyadh	113 (46.3%)	82 (55.0%)	0.093
Outside Riyadh	131 (53.7%)	67 (45.0%)	
Most common gaming platform^†^			
Phone	174 (71.3%)	117 (78.5%)	0.114
Tablet	54 (22.1%)	31 (20.8%)	0.757
Personal computer	65 (26.6%)	29 (19.5%)	0.106
PlayStation	150 (61.5%)	61 (40.9%)	<0.001*
Xbox	13 (5.3%)	08 (5.4%)	0.985
Nintendo	16 (6.6%)	04 (2.7%)	0.090
Number of days spent in a week playing video games			
Every day	178 (73.0%)	70 (47.0%)	<0.001*
Not every day	66 (27.0%)	79 (53.0%)	
Number of hours spent in a day playing video games			
≤3 hours	76 (31.1%)	94 (63.1%)	<0.001*
>3 hours	168 (68.9%)	55 (36.9%)	
Playing multiplayer games regularly (online games)			
Yes	235 (96.3%)	117 (78.5%)	<0.001*
No	09 (03.7%)	32 (21.5%)	
Type of games usually played			
Shooter games	107 (43.9%)	47 (31.5%)	0.024*
Sports and adventure games	90 (36.9%)	59 (39.6%)	
Action and other games	47 (19.3%)	43 (28.9%)	
Where do you play usually?			
Alone in the room	157 (64.3%)	76 (51.0%)	0.009*
In the presence of the family	87 (35.7%)	73 (49.0%)	
Do the parents supervise the games you play?			
Yes	111 (45.5%)	81 (54.4%)	0.088
No	133 (54.5%)	68 (45.6%)	
Does the child or adolescent mimic the games in real life like doing impulsive movements or saying bad words?			
Yes	77 (31.6%)	26 (17.4%)	0.002*
No	167 (68.4%)	123 (82.6%)	

As per the multivariate regression model performed (Table 6), the chance of being addicted to playing video games among adolescents could likely decrease by at least 42% (AOR: 0.582; 95% CI: 0.344-0.985; p=0.044). Respondents who were playing every day were at a 1.9-fold higher risk of being addicted to video games than those who were not playing every day (AOR: 1.996; 95% CI: 1.206-3.303; p=0.007). Also, respondents who were playing video games for more than three hours per day were at a 2.2-fold higher risk of being addicted to it than those who were playing fewer hours (AOR: 2.199; 95% CI: 1.338-3.614; p=0.001). Respondents who played multiplayer games had at least 4.2 times higher odds to have game addiction (AOR: 4.228; 95% CI: 1.808-9.890; p=0.001). Other variables included in the model were not significantly associated with game addiction after adjusting for the regression model, including gender, playing games on PlayStation, type of games usually played, gaming patterns, and mimicking games in real life (p>0.05).

**Table 6:** Multivariate regression analysis to determine the independent significant factors associated with game addiction (n=393) *Significant at p<0.05 level AOR: adjusted odds ratio; CI: confidence interval

Factor	AOR	95% CI	P-value
Age group			
12–13 years	Ref.		
14–16 years	0.582	0.344–0.985	0.044*
Gender			
Male	1.357	0.812–2.269	0.244
Female	Ref.		
Playing games on PlayStation			
Yes	1.575	0.945–2.622	0.081
No	Ref.		
Number of days spent in a week playing video games			
Every day	1.996	1.206–3.303	0.007*
Not every day	Ref.		
Number of hours spent in a day playing video games			
≤3 hours	Ref.		
>3 hours	2.199	1.338–3.614	0.002*
Playing multiplayer games regularly (online games)			
Yes	4.228	1.808–9.890	0.001*
No	Ref.		
Type of games usually played			
Shooter games	Ref.		
Sports and adventure games	0.745	0.392–1.415	0.369
Action and other games	0.745	0.403–1.379	0.349
Where do you play usually?			
Alone in the room	1.503	0.906–2.493	0.115
In the presence of the family	Ref.		
Does the child or adolescent mimic the games in real life like doing impulsive movements or saying bad words?			
Yes	1.585	0.908–2.767	0.105
No	Ref.		

## Discussion

This study investigated links between video game addiction and depression, anxiety, and attention deficit among adolescents (aged between 12-16 years) in Saudi Arabia. The findings of this study suggest that 62.1% of the subjects are addicted to playing video games with 63.1% reported playing video games daily for a minimum of five hours or more (25.2%). The addiction to video games in our population can be compared to that among American adolescents [14]. According to reports, 93% of adolescents play video games regularly irrespective of age, gender, and socioeconomic levels with an average of 6.3 hours per week spent on playing. However, Saquib et al. [2] showed that only 16% were addicted to video gaming but as much as 54% of the adolescents demonstrated psychological distress due to excessive video game playing. Several studies have established a link between mental disorders and using electronic gadgets [15,16,17,18]. This is in line with our study, as we found a positive correlation between the score of game addiction and the scores of depression, anxiety, and inattention, indicating that more time consumed playing video games is directly correlated with increased symptoms of mental disorders including depression, anxiety, and attention deficit among Saudi children and adolescents. In contrast, a study conducted among university students found that there was no significant difference in anxiety levels or daytime sleepiness between expert and non-expert video gamers and there were only minimal to mild anxiety levels demonstrated by the subjects. The rise of mental health disorders associated with excessive video game playing among the young population is quite alarming and requires interventions in the form of raising awareness and educational measures on the part of health authorities.

Literature suggests that children with inattention are more susceptible to gaming addiction due to neuropsychological issues [19]. According to Masi et al, [18], children with inattention exhibit more addictive behaviors concerning video games and prolonged periods of use than normal children. In our study, there was a direct relationship between video game addiction and ADHD scale scores. Despite research highlighting some of the damaging effects of video games, there are certain potential benefits to children from using video games, which should not be dismissed [20]. Parents should take advantage of this and teach children to make better use of video games, such as introducing educational games and other alternative video games that could enhance the motor development of their children. Ultimately, the guidance of parents in teaching their children to be responsible gamers is vital to preventing potential psychological disorders associated with video game addiction.

Our findings suggest that age, gender, playing games on PlayStation, playing extended periods daily, playing multiplayer games regularly, playing shooter-type games, playing alone, and children or adolescents mimicking games in real life were the important variables in terms of video game addiction; however, in our multivariable regression analysis, only age, playing for an extended period of time, and playing multiplayer games regularly remained significant with regard to video game addiction. In a study by Schou Andreassen et al. [21], age was inversely associated with technology addiction, and while males tended to be more addicted to video games, females tended to be more addicted to social media. This is almost consistent with our findings, as we detected that increasing age was associated with a decrease in video game addiction, and males exhibited an increased risk of addiction to video games. A study conducted In Iran [22] reported that female teenage students with older mothers tend to play video games more often than teenagers with younger mothers and showed disturbing mental behavior. It is interesting to see how sociodemographic variables can influence video gaming addiction. Further investigation into this phenomenon is warranted.

Parent supervision while children are playing video games is necessary to monitor what types of games they are playing and the time spent on this activity. In our study, although nearly half of the parents (48.9%) reported supervising their children during video game playing, 59.3% of children reported playing alone in the room, and 26.2% of them stated that they tried to mimic the game in real life, such as imitating the actions of the character they used in the games as well as copying what the character said in the game, including using age-inappropriate language. The findings of a recent literature review [19] showed that playing computer games that feature excessive violence increases the risk potential for aggressive behavior in the setting of personality traits with violent thoughts and behaviors; early intervention with behavioral scripts could be beneficial in such scenarios. Another review of articles involving 101 studies reported that the influence of video games may result in increased aggression, decreased prosocial behavior, decreased academic performance, and depression, while attention deficit symptoms are less pronounced [17]. Concerned agencies should prioritize raising awareness among parents and children regarding the possible harmful effects of excessive video game playing on children's health and psychosocial functioning. 

Limitations of the study

One limitation of this study is that we used screening scales rather than clinical interviews to diagnose depression, anxiety, and inattention. This may have resulted in clinical bias. Another limitation is that we did not address the duration of gaming addiction and its association with depression, anxiety, and inattention.

## Conclusions

Our findings reveal that there is a high prevalence of video game addiction among children and adolescents in Saudi Arabia. Excessive video game playing can negatively influence mental health, leading to issues such as anxiety, depression, and inattention. Furthermore, younger males who engage in gaming for prolonged periods of time regularly are more likely to exhibit video game addiction when compared to the rest of the population. The findings of this study highlight the need for educational interventions to curb excessive video game playing and its potentially harmful effects on children's and adolescents' mental and physical health. Further research is needed to gain deeper insights into the prevalence of video game addiction and its effect on the mental health of young people in the region.
